# The effects of pregabalin and adductor canal block on postoperative pain in arthroscopic anterior cruciate ligament reconstruction

**DOI:** 10.3906/sag-1906-66

**Published:** 2020-02-13

**Authors:** Fatma KAVAK AKELMA, İlkay BARAN AKKUŞ, Savaş ALTINSOY, Derya ÖZKAN, Jülide ERGİL

**Affiliations:** 1 University of Health Sciences, Anaesthesiology and Reanimation Clinic,Dışkapı Yıldırım Beyazıt Training and Research Hospital, Ankara Turkey

**Keywords:** Pregabalin, adductor canal block, postoperative pain

## Abstract

**Background/aim:**

To determine the effectiveness of pregabalin and adductor canal block on opioid consumption, postoperative pain, and fast-tracking.

**Materials and methods:**

A total of 51 American Society of Anaesthesiologists (ASA) classification I–II patients aged 18–70 years who were scheduled to undergo elective anterior cruciate ligament reconstruction were included in the study. Patients were randomized into groups P, A, and C. Patients in group P (n = 16), received 150 mg of preoperative oral pregabalin, patients in group A (n = 17) received postoperative adductor canal blockade, and patients in group C (n = 18) received neither adductor canal block nor pregabalin. Surgeries were performed under spinal anaesthesia with hyperbaric bupivacaine following monitorization. Demographic data along with block features, hemodynamic data, mean opioid consumption, numerical rating scale score, White’s fast-track score, and postoperative adverse effects were recorded.

**Results:**

Fifty-seven patients were enrolled in the study, and 6 patients were excluded from the study; the data of 51 patients were included in the final analyses. Demographic characteristics and hemodynamic data were similar between the 3groups. Postoperative opioid consumption was significantly lower in groups A and P compared with group C (group P = 178.75 mg, group C = 318.61 mg, group A = 236.47 mg; P < 0.05). The regression of sensory block was significantly slower in group P (P < 0.05). The first analgesic requirement was earlier in group C than in groups P and A (P < 0.05). Patients in group P had higher fast-track scores at 8 h and 12 h compared with group C (P < 0.05); however, group A fast-track scores were similar to those of the other 2groups (P > 0.05). The rate of postoperative adverse effects was similar between the groups (P > 0.05).

**Conclusion:**

Preoperative pregabalin (150 mg) reduced postoperative opioid consumption as much as adductor canal block in patients undergoing anterior cruciate ligament reconstruction. The first analgesic requirement was earlier in group C than in groups P and A. In addition, pregabalin can prolong the duration of spinal sensory block and shorten the time required to achieve high fast-tracking scores. We recommend the use of both methods as a part of multimodal analgesia.

## 1. Introduction

Repair of the anterior cruciate ligament (ACL) is one of the most commonly performed outpatient arthroscopic procedures [1,2]. Optimal analgesia facilitates early rehabilitation and mobilization, improves functional recovery, reduces postoperative morbidity, and increases patient satisfaction after anterior cruciate ligament reconstruction (ACLR) [1,3]. Multimodal analgesia using opioids and a variety of other analgesics is recommended for the management of acute pain after knee surgery [4].

Pregabalin is a structural analogue of γ-aminobutyric acid (GABA) acting on the α2δ subunit of voltage-dependent calcium channels. Pregabalin is frequently used as a part of neuropathic and postoperative pain management [4]. Previous studies demonstrated that preoperative pregabalin reduced opioid consumption during the 24-h postoperative period in patients who received spinal or general anaesthesia [5–11]. 

The adductor canal block (ACB) has recently gained popularity as an alternative to femoral nerve blockade due to the reduced incidence of quadriceps muscle weakness [2,12]. Several neural structures traverse the canal including the saphenous nerve and its infrapatellar branch, the nerve to the vastus medialis, the posterior branch of the obturator nerve, and in some cases, the medial cutaneous nerve and the anterior branch of the obturator nerve. With the exception of the nerve to the vastus medialis, these branches provide sensory innervation of the anterior and medial knee. ACB combined with a local anaesthetic infusion within the canal not only provides efficient analgesia but also contributes to maintaining lower extremity motor functions following knee surgery [12].

The primary objective of the current study was to evaluate the effect of a single dose of oral pregabalin and ACB on opioid consumption in patients undergoing arthroscopic ACLR. The secondary objectives were to compare its effectiveness on postoperative pain, spinal block characteristics, and fast-tracking. We hypothesized that this treatment would reduce postoperative opioid consumption of oral pregabalin and ACB when compared with a control group.

## 2. Materials and methods

The trial was approved by the Ethical Committee of the Ministry of Health, Dışkapı Yıldırım Beyazıt Training and Research Hospital, Ankara, Turkey (ethical committee 29.01.2018, no.: 45/17). The study was conducted at the University of Health Sciences, Dışkapı Yıldırım Beyazıt Training and Research Hospital, between June 2018 and August 2018.

A single-centre, prospective, randomized, patient- and assessor-blinded, placebo-controlled study was performed. All patients were informed, and written informed consent was obtained from the patients. 

Patients who were listed to undergo elective unilateral ACLR were screened for inclusion in the study. The inclusion criteria were as follows: elective unilateral arthroscopic reconstruction of the ACL, age 18–70 years, and American Society of Anaesthesiologists (ASA) classification I–II. The exclusion criteria were as follows: refusal to participate, inability to provide informed consent, contraindication to ACB (local infection, local anaesthetic allergy, and coagulopathy), contraindication to neuraxial anaesthesia (patient refusal, coagulopathy or bleeding diathesis, skin infection at the lumbar area, increased intracranial pressure, and allergy to local anaesthetics), peripheral neurologic dysfunction or neuropathy, surgery under general anaesthesia, body mass index (BMI) greater than 45 kg/m2, known allergy to any medicine, history of drug or alcohol abuse, use of opioids or sedative medications, history of psychiatric conditions, and pregnancy or lactation.

**Figure 1:**
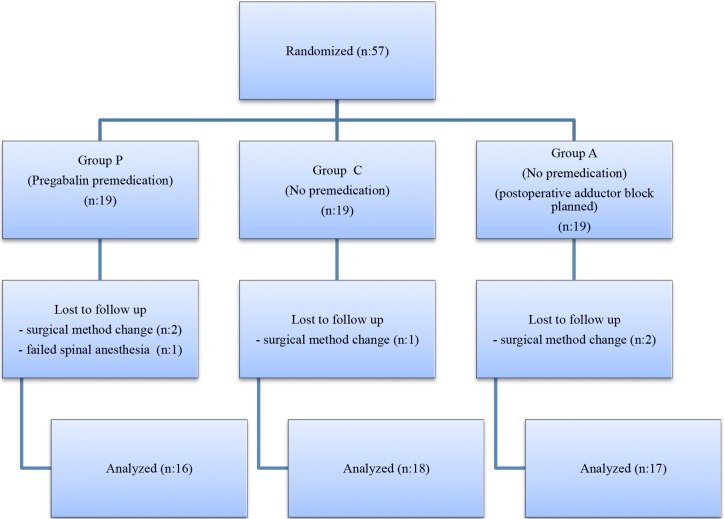
Flow chart.

Preoperative visits by an anaesthesiologist were conducted for all patients, and patients were instructed in the use of the numeric rating scale (NRS) for pain assessment (0 = no pain; 10 = worst pain imaginable) and a system for patient-controlled analgesia (PCA). Patients were randomly divided into 3groups after recruitment using a computer-generated list. The patient was blinded to the treatment, and all records were recorded by an anaesthesiologist blinded to group allocation (Figure 1).

One h before anaesthesia induction, 150 mg pregabalin capsules (Lyrica 150 mg capsule, Pfizer, İstanbul, Turkey) were administered to group P patients. During the same period, capsules containing a placebo were administered to group C and group A patients. Placebo capsules were prepared by the pharmacy and were identical to the relevant trial drugs in size, shape, colour, and weight and were tasteless. Premedication was not applied to any of the patients. The electrocardiogram, blood pressure, and peripheral oxygen saturation were monitored upon patient entry into the operating room and subsequently every 5 min. Intravenous access was established using a 20-G intravenous cannula, and each patient was preloaded with Ringer’s lactate solution (15 mL/kg). Spinal anaesthesia was administered in the sitting position in the L4-L5 space with 2.5 mL of 0.5% hyperbaric bupivacaine using a midline approach with a 25-G Quincke needle. Oxygen was administered to all patients via facemask at 2–4 L/min. If the patient experienced either a decrease in systolic blood pressure <30% from the baseline value or a mean arterial blood pressure <60 mm Hg, repeated doses of intravenous ephedrine (10 mg) were administered. Routine intraoperative sedation was not provided. The sensory block was evaluated every 2min by loss-of-pinprick discrimination, and the motor block was evaluated using the Bromage scale [13]. Onset times for sensory and motor blocks were recorded. Surgical intervention was initiated when the block reached the T10 level. The onset time of sensory block at the L1-T10 level, the highest level of sensory block, duration of sensory block, time for 2-segment regression of sensory block, and time for regression to Bromage 2 were recorded. 

All patients subsequently underwent arthroscopically assisted ACL reconstructions with bone-tendon-bone (BTB) autograft. Patients were blocked under ultrasound guidance after surgery. Surgical bandages were applied before surgical closure and were positioned with mild external rotation of the surgical limb. Under sterile conditions, the femoral artery, vein, and accompanying nerve branches were located using ultrasound (HFL 38X/13–6 MHz; SonoSite M-Turbo ultrasound machine; SonoSite Inc., Bothell, WA, USA) halfway between the anterior superior iliac spine and the patella. The patient ultrasound monitor and block were isolated such that the application area was not seen. After local anesthesia in group A, an 18-G 10 cm 50 mm stimulator needle (Echoplex, Vygon, Ecouen, France) was inserted under ultrasound guidance in the plane 30° off of a short-axis view until it was visualized next to the nerve(s) and artery under the fascia of the sartorius muscle [14]. Then, 10 mL of 0.25% bupivacaine with 5 μg/mL epinephrine was administered in fractionated doses via ultrasound guidance by the same anaesthesiologist [12]. Group P and group C patients were placed in similar positions and simulated adductor blocks were performed with an ultrasound probe. Then, in group P and group C, a sham subcutaneous injection of 0.5 mL of sterile normal saline was administered at the ACB site using ultrasound guidance with transducer pressure intended to simulate a real block procedure. Standard surgical bandages were then applied to the operated knees of all patients including the puncture part of the needle. 

Upon discharge from the postanaesthesia care unit, all patients received a standard postoperative multimodal regimen of dexketoprofen trometamol (50 mg every 12 h) and a tramadol intravenous PCA (10 mg bolus with a 10 min lockout period) device (CADD-Legacy PCA pump, Smiths Medical, USA) for 24 h. Postoperative pain was assessed by the patient using the NRS and WFTSS [15] (Appendix 1) at 1, 4, 8, 12, and 24 h after surgery, and results were recorded by a blinded researcher. Patients with a NRS score of 4 or more received 50 mg of tramadol intravenously. Time of the first request for postoperative analgesia, number of injections, total amount of tramadol consumed by the PCA device after 24 h, and patient demand were recorded. All assessments were recorded by a blinded researcher.

 The incidence of opioid-related side effects, nausea and vomiting, dry mouth, urinary retention, pruritus, pregabalin-related side effects, confusion, headache, diplopia, and dizziness were defined as present when at least one episode was noted in the first 24 h after surgery.

G*Power version 3.1.9.2 (Franz Faul, Edgar Erdfelder, Albert-Georg Lang, and Axel Buchner, 2006, 2009) was used to perform a priori sample size calculations based on pilot data on postoperative 24 h tramadol consumption [16]. In the preliminary study, which included 10 patients, tramadol consumption (mean ± SD) was 180 ± 59.31 in the pregabalin group and 246 ± 74.12 in the adductor group. A 25% decrease in tramadol consumption was detected in group P compared to group A. Accordingly, the number of patients required in each group was determined with α-error = 0.05 at 85% power, at least 16 patients per group. In the current study, 19 patients were included for analysis in each group to account for potential dropout.

Data were analysed using SPSS software version 24 (IBM Corp., Armonk, NY, USA). The normality of the distribution of continuous variables was evaluated using one sample Shapiro–Wilk test. Patient demographics and characteristics were expressed as number and percentage, median [interquartile range (IQR)], and mean [standard deviation (SD)] and were analysed using the chi-square test for categorical variables and the independent t-test for normally distributed continuous variables. Mann–Whitney U test and Kruskal–Wallis were applied for comparisons of nonparametric and nonnormally distributed data. Nominal data were analysed by Pearson’s chi-square or Fisher’s exact test where appropriate. The corrected Bonferroni test was used for multiple comparisons. P-values of <0.05 were considered statistically significant in each test.

## 3. Results

Fifty-seven patients were enrolled in the study. Patients were randomly assigned to 1 of 3 groups. Five patients had a surgical method change, and one patient received general anaesthesia due to failed spinal anaesthesia. Consequently, 51 patients (group P, n = 16; group C, n = 18; group A, n = 17) were included in the study (Figure 1). 

**Figure 2:**
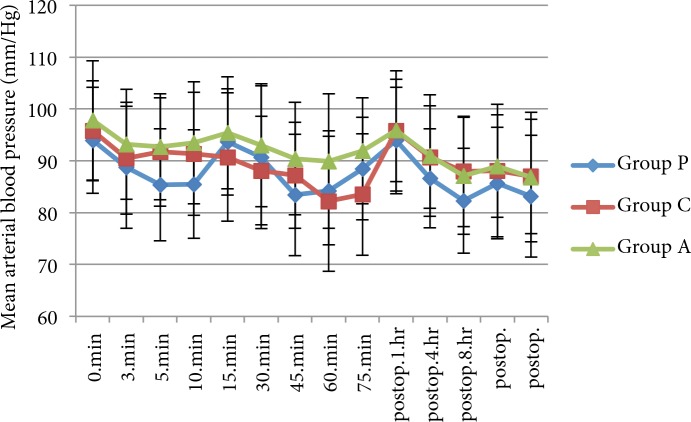
Mean blood pressure. Data were expressed as mean ± SD. Postop = postoperative.

**Figure 3:**
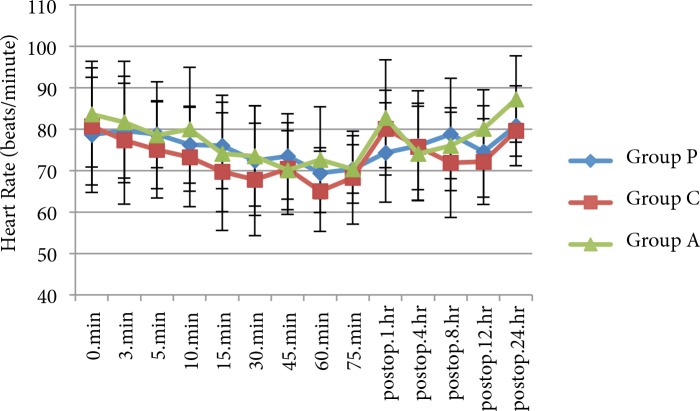
Heart rate. Data were expressed as mean ± SD. Postop = postoperative.

Age, sex, BMI, ASA status, duration of surgery (min), duration of anaesthesia (min), and intraoperative ephedrine and atropine consumption were similar between the groups (P > 0.05) (Table 1). The 3groups were similar in terms of mean heart rate and mean arterial blood pressure (P > 0.05) (Figures 2 and 3). T10 sensory block, time to Bromage 3 block, mean maximal sensory level, time to regression to Bromage 2, and time to resolution of motor blockade were similar between the groups (P > 0.05) (Table 2). Time to 2-segment regression of sensory block was longer in patients who received pregabalin compared with groups A and C (P < 0.05), as shown in Table 2. 

**Table 1:** Demographic data.

Variables	Group P(n = 16)	Group C(n = 18)	Group A(n = 17)	P-value
Age (year)	29.50 ± 9.49	33.27 ± 14.06	28.76 ± 8.26	0.434
Body mass index (kg/m2)	27.59 ± 3.90	27.45 ± 5.69	26.09 ± 2.89	0.549
Sex (female/male) (n)	2/14	3/15	3/14	0.690
ASA status (I/II)(n)	9/7	9/9	10/7	0.864
Duration of surgery (min)	80.25 ± 35.20	75.50 ± 26.94	80.76 ± 24.13	0.839
Duration of anaesthesia (min)	90.68 ± 33.80	84.94 ± 26.71	90.05 ± 23.94	0.807
Intraoperative ephedrine (mg)	0 (0-20)	0 (0–20)	0 (0–10)	0.632
Intraoperative atropine (mg)	0 (0-0.5)	0 (0–0.5)	0	0.391

The data are presented as mean ± SD, median (IQR).

**Table 2:** Onset time and duration of sensory and motor blocks.

Variables	Group P(n = 16)	Group C(n = 18)	Group A(n = 17)	P-value
Time to T10 sensory block (min)	6.43 ± 3.61	5.44 ±1.94	5.29±1.86	0.389
Time to Bromage 3 block (min)	9.37 ± 4.44	9.33±3.80	9.17±3.28	0.988
Mean of the maximal sensory level (dermatome)	8 (6–10)	6 (6–10)	6 (5–10)	0.537
Time for 2-segment regression of sensory block (min)	70 (60–112.5)a	47.5 (45–63.75)	50 (45–60)	0.008
Time for regression to Bromage 2 (min)	120 (75–180)	120 (120–180)	120 (120–130)	0.678
Time to resolution of the motor blockade (min)	240 (180–292.5)	235 (207.5–255)	240 (200–290)	0.824

The data are presented as mean ± SD, median (IQR). aAccording to the Bonferroni correction, group P was different than groups C and A (P = 0.020 and P = 0.039, respectively).

**Figure 4:**
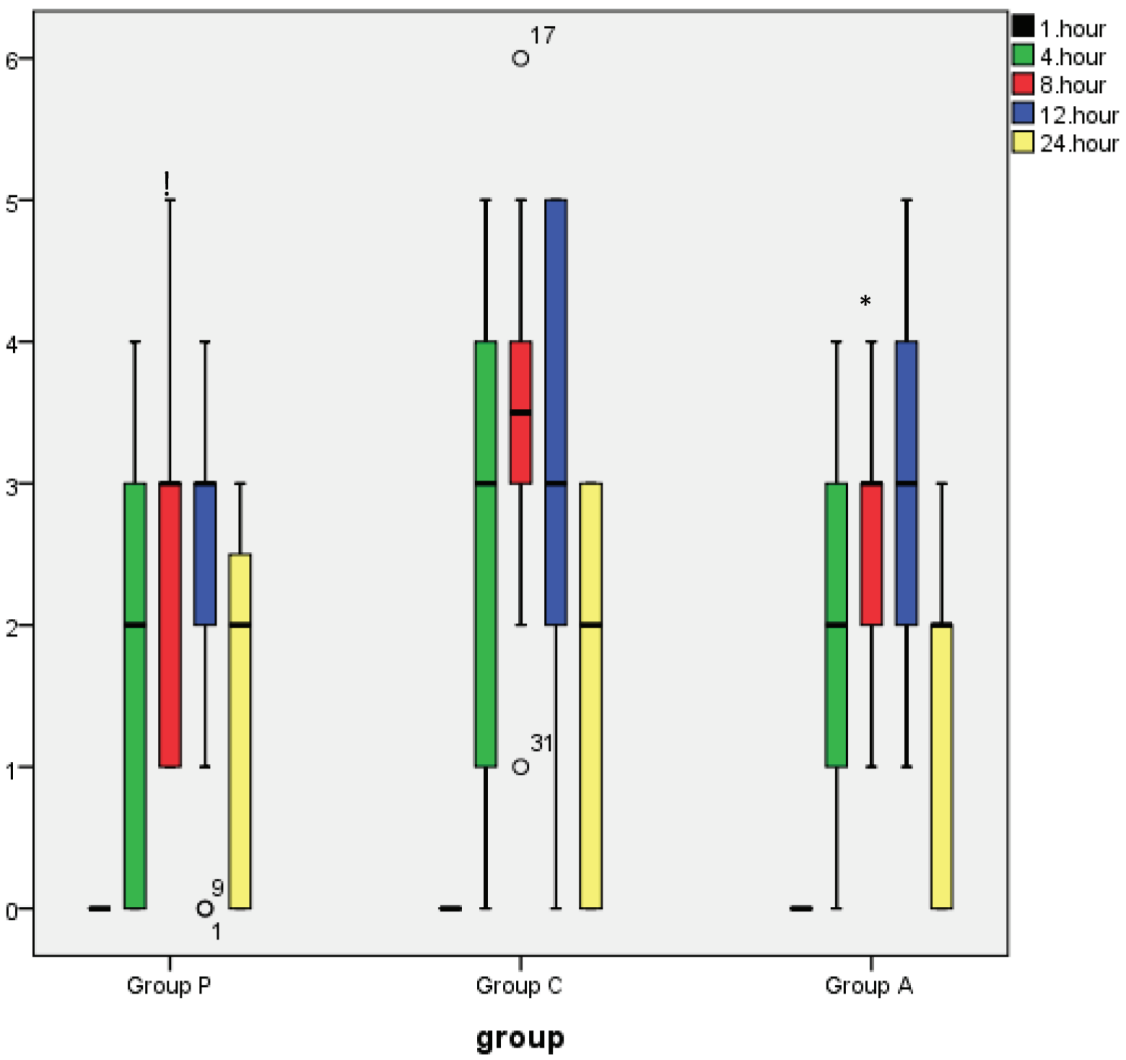
Rest NRS score. These box plots display the median and 25th and 75th percentiles of the NRS (pain at rest) rating at different time points; asterisk = extreme outliers, open circles = slight outliers. *Significant compared to the control group (P = 0.040), and Significant compared to the control group (P = 0.049).

**Figure 5:**
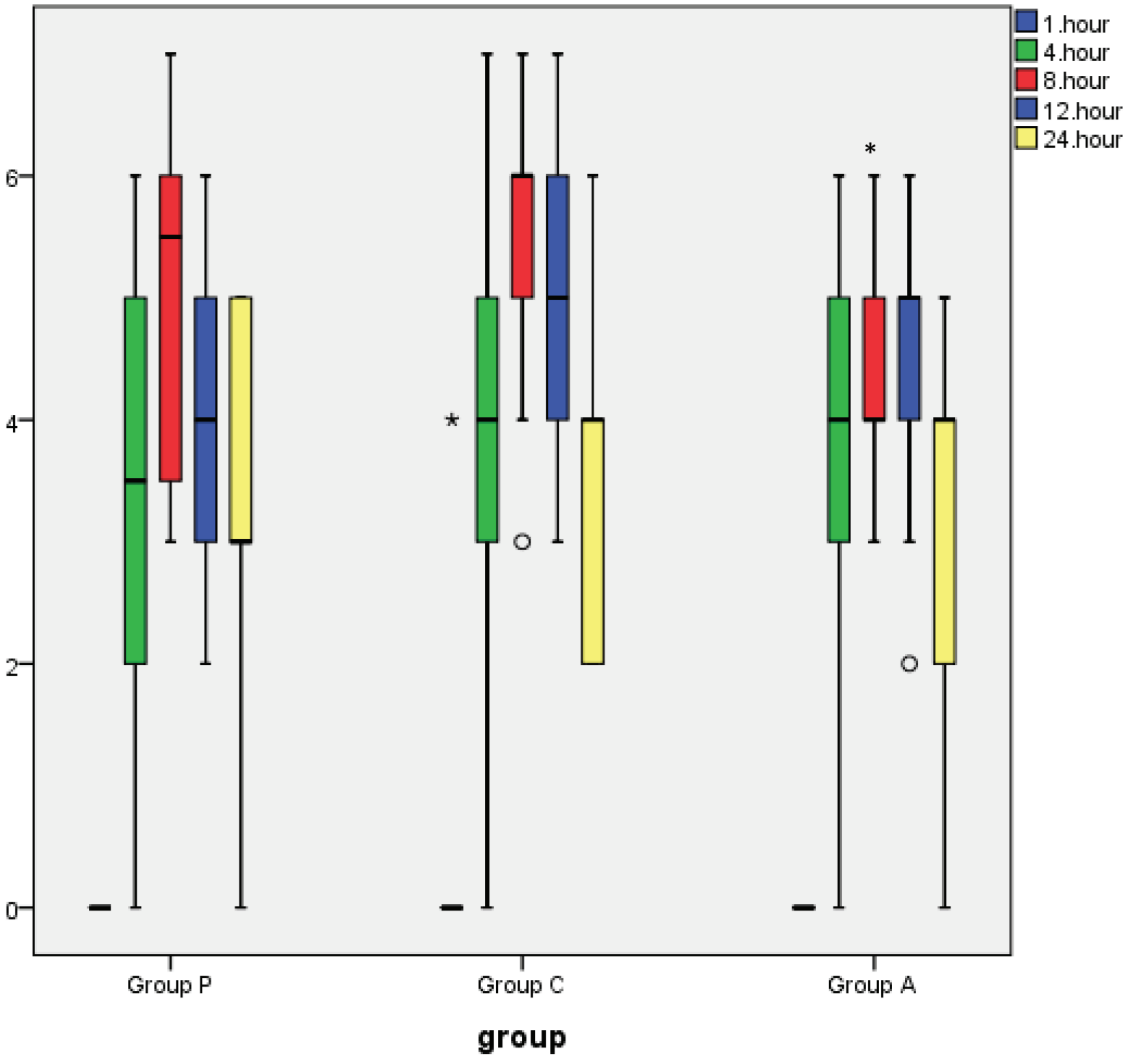
Dynamic NRS score. These box plots display the median and 25th and 75th percentiles of the NRS rating at different time points; asterisk = extreme outliers, open circles = slight outliers. and Significant compared to the control group (P = 0.022).

Tramadol consumption was lower in groups P and A compared with group C (group P = 178.75 mg, group C = 318.61 mg, group A = 236.47 mg; P < 0.05) and was similar between group P and group A (P > 0.05), as shown in Table 3. The first analgesic requirement was earlier in group C than in groups P and A (P = 0.001), and no obvious differences were observed between group A and group P (Table 3). The number of PCA demands, the number of patients who required rescue analgesics, and the amount used were similar between the groups (P > 0.05) (Table 3). The rest NRS score at 8 h was significantly lower in groups P and A compared with group C (P = 0.040, P = 0.049, respectively), and the rest NRS score was similar between group P and group A (Figure 4). The dynamic NRS score at 8 h was lower in group A than in group C and was similar between group P and group A (P = 0.022) (Figure 5). The rest and dynamic NRS scores were similar in the other time interval (Figures 4 and 5).

**Table 3:** Postoperative features.

Variables	Group P(n = 16)	Group C(n = 18)	Group A(n = 17)	P-value
Tramadol consumption 24 h (mg)	178.75 ± 65.40b	318.61 ± 127.89a	236.47 ± 80.69b	0.001
Number of PCA demand (n)	27 (17–51)	51 (38–84)	40 (23–65)	0.220
Total rescue analgesic consumption (mg)	0 (0–50)	25 (0–100)	0 (0–50)	0.174
Number of rescue analgesics (n)	6	9	5	0.469
Time of first analgesia requirement (min)	386.25 ± 47.59	272.77±45.21c	343.52±66.51	0.001

The data are presented as mean ± SD, median (IQR). aAccording to the Bonferroni correction, group C was different than groups P and A ( P = 0.001 and P = 0.046, respectively). bAccording to the Bonferroni correction, group P was similar to group A (P = 0.277). cAccording to the Bonferroni correction, group C was different than groups P and A (P = 0.001 and P = 0.002, respectively).

**Figure 6:**
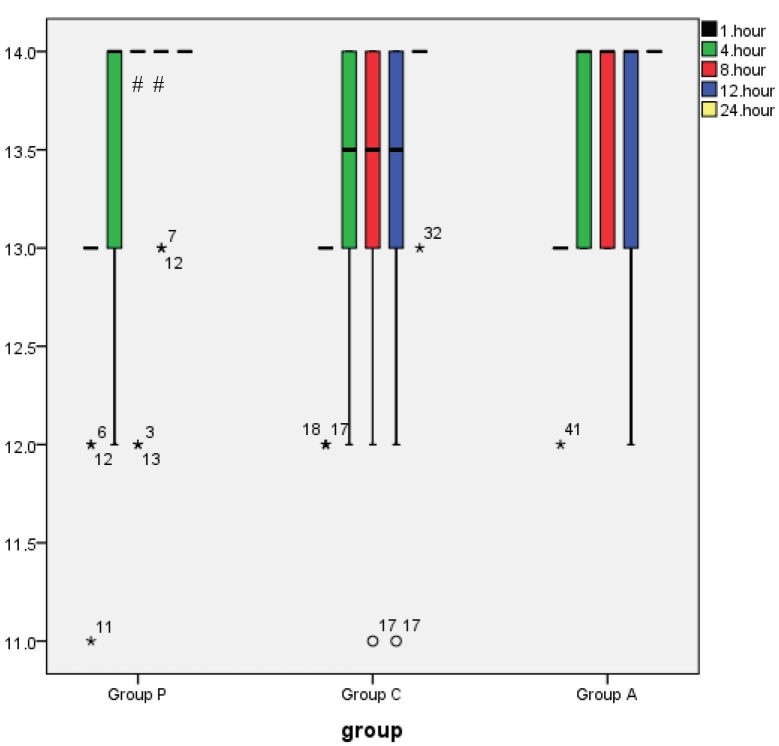
These box plots display the median and 25th and 75th percentiles of White fast-track scores rating at different time points; asterisk = extreme outliers, open circles = slight outliers. #Significant compared to the control group (P < 0.05).

The fast-tracking score was higher in group P than in group C at 8 h and 12 h and was similar in the other time intervals (P < 0.005) (Figure 6). The fast-tracking score was similar between group P and group A in the all-time interval (P > 0.05) (Figure 6). In addition, the fast-tracking score was similar between group A and group C in the all-time interval (P > 0.05) (Figure 6). 

Two patients in each group developed nausea and vomiting, and 1 patient in group P had drowsiness. Itchiness was observed in 1 patient in group A, urinary retention in 3 patients in group A and 1 patient in each of the groups P and C. None of the patients reported headache, dry mouth, voiding difficulties, diplopia, or confusion.

## 4. Discussion

In this study, patients who received pregabalin and ACB had significantly lower consumption of opioids than the control group. The first analgesic requirement was earlier in group C than in groups P and A. In addition, the regression of sensory block was significantly slower in the pregabalin group. Finally, in group P the number of patients reaching the maximum total fast-track score at the 8th h was higher than in the control group. 

Although ACLR is less invasive in nature than conventional knee surgery, patients can still experience moderate-to-severe postarthroscopic pain. Inadequate postoperative analgesia can delay discharge, increase unplanned admission and readmission after discharge, delay functional recovery, and reduce patient satisfaction with ACLR [1,3,12]. Although opioids have side effects, they still have an important role in postoperative pain management [6]. To reduce opioid-related side effects, multimodal analgesia, including local infiltration analgesia, NSAIDs, peripheral nerve blocks, and other adjuncts such as pregabalin have been suggested [2]. For these reasons, we used ACB from peripheral blocks and pregabalin from adjuvant agents for the management of postoperative analgesia.

Pregabalin is an analgesic anticonvulsant with anxiolytic effects. It is frequently used in the treatment of chronic neuropathic pain but is increasingly used in the treatment of acute pain [17]. As a part of multimodal analgesia, pregabalin reduces opioid consumption and relieves postoperative pain in patients undergoing knee surgery [11]. Likewise, ACB is applied to reduce postoperative pain and opioid consumption in patients undergoing knee surgery [12]. There is not yet proof of superiority among the methods. Each method has adverse effects. For example, pregabalin may cause dose-dependent adverse effects including somnolence, sedation, and dizziness [9]. Conversely, ACB can cause adverse effects such as prolonged motor block and quadriceps weakness [18].

The effectiveness of a single preoperative dose of pregabalin on postoperative analgesia has been explained using an electrical hyperalgesia model. This theory showed that pregabalin reduced central sensitization and exerted antihyperalgesic effects during, and immediately following, surgery [19]. Different doses of pregabalin (75, 150, 300, 600 mg, etc.) are preferred in acute pain, but higher doses have been associated with more adverse effects. A few studies have shown that a single preoperative oral dose of pregabalin (150 mg) can reduce postoperative pain and adverse effects in patients undergoing orthopaedic surgeries, as well as other surgeries [4,10,11]. In this study we also used the lowest effective dose of preoperative pregabalin (150 mg), which was similarly effective as doses used in the literature.

In a study conducted on patients undergoing spinal surgery, Fujita et al. [7] found that morphine consumption and postoperative pain scores decreased in the pregabalin group. In a study conducted on patients who underwent total knee arthroplasty and received either adductor canal blockade or saline, Nader et al. [12] found that ACB effectively reduced pain and opioid requirements in the postoperative period. In this study, opioid consumption was similar between the pregabalin and adductor groups and was significantly lower in these groups compared to the control group during the 24-h postoperative period. Rest and dynamic NRS scores were similar in all 3 groups with the contribution of spinal anaesthesia up to the 8th h. Rest and dynamic NRS scores were lower in both pregabalin and ACB groups at the 8th h compared to the control group. This may be due to the relatively long half-life of pregabalin with the consequent prolongation in sensory block [20]. 

Studies assessing sensory and motor block characteristics following the use of a single dose of pregabalin in patients receiving regional anaesthesia are limited. Cegin et al. [21] compared the pregabalin and control group results in patients undergoing upper extremity bone surgery under infraclavicular block. They found that sensorial block termination durations were prolonged in the pregabalin group, but the duration of the motor block did not change. Park et al. [22] compared the effectiveness of pregabalin in patients under spinal anaesthesia and reported that a single dose of preoperative pregabalin significantly prolonged the duration of sensory-motor blockade and 2-segment sensory regression and reduced total opioid consumption compared to placebo. Kampitak et al. [23] performed postoperative single-injection ACB or local infiltration analgesia in patients undergoing total knee arthroplasty under spinal anaesthesia and found no difference between the groups in terms of time to first request for analgesia. Total morphine consumption was, however, lower in the ACB group. In this study, it was found that the time to 2-segment regression of sensory block was longer in patients who received pregabalin compared to the ACB and control groups. The duration of motor block and time to regression of motor block was similar between the groups. This study showed that oral pregabalin (150 mg) administered 1 h before spinal anaesthesia prolongs only the sensory blocks induced by spinal bupivacaine anaesthesia. The mechanisms by which pregabalin premedication prolongs sensory blocks using local anaesthetics in spinal anaesthesia are not clear. Evoked pain during movement is enhanced by central neuronal sensitization [22,24], and the permanent pregabalin effects observed in our study may have been caused by preoperative pregabalin preventing central nervous system sensitization. In our opinion, although groups differed with regards to 2-segment regression of sensory block, combined peripheral nerve block in group A may have prolonged the time to first request for analgesia, reducing total opioid consumption in this group and, eventually, leading to a similarity in total opioid consumption between the 2 groups. 

Effective reduction of pain in the postoperative period in knee surgery has contributed to the rapid disappearance of undesirable motor blockade and shorter discharge time [12]. Nader et al. [12] compared ACB and a control group and found that ACB provides better analgesia and shorter discharge time. They suggested that ACB should be part of the fast-track protocol in patients undergoing total knee arthroplasty. In the current study we did not observe superiority in fast-tracking in group A compared to the control and pregabalin groups. In group P, however, the number of patients reaching the maximum total fast-track score at the 8th h was higher than in the control group. In other words, patients in the pregabalin group could be ready for discharge from the 8th h. According to the routine postoperative discharge protocol in our hospital, patients are not discharged during the first 24 h. Therefore, we could not discharge the patients according to their fast-tracking scores.

Several studies have shown that postoperative pain could persist for 2 to 6 weeks after discharge, and this could limit patient participation in daily activities [4]. Additionally, poorly controlled postoperative pain may lead to chronic pain [25]. Although studies have shown that preoperative pregabalin provides longer analgesia [26], in the current study we did not follow up on chronic pain. A potential limitation of this study is the relatively short postoperative follow-up period. Another limitation is that the volume (10 mL) used for ACB was lower than volumes used in clinical practice (i.e. 15 mL, 20 mL vs. 25 mL), because higher volume may influence the incidence of quadriceps muscle weakness. A comparison of different doses of bupivacaine may be performed in subsequent studies.

In conclusion, preoperative pregabalin provides postoperative analgesia that is as effective as ACB. In addition, pregabalin can shorten the time to high fast-tracking scores in patients undergoing ACLR. The pregabalin group was superior in terms of sensory block duration compared to the ACB and control groups. However, this advantage did not differ between pregabalin and ACB groups in terms of total postoperative opioid consumption. We recommend the adoption of both methods as a part of multimodal analgesia.

## Authorship contributions

Surgical and medical practices: F.K.A., İ.B., S.A.; concept: F.K.A., J.E.; design: F.K.A., D.Ö., J.E.; data collection or processing: F.K.A., S.A., İ.B.; analysis or interpretation: F.K.A., S.A, D.Ö.; literature search: F.K.A, İ.B.; and writing: F.K.A., S.A., D.Ö.

## Acknowledgement

The authors declared that this study received no financial support. 

## Informed Consent

Consent form was filled out by all participants.
